# Weekly paclitaxel plus ramucirumab versus weekly nab-paclitaxel plus ramucirumab for unresectable advanced or recurrent gastric cancer with peritoneal dissemination refractory to first-line therapy—the P-SELECT trial (WJOG10617G)—a randomised phase II trial by the West Japan Oncology Group

**DOI:** 10.1186/s12885-020-07047-1

**Published:** 2020-06-12

**Authors:** Kenro Hirata, Yasuo Hamamoto, Masahiko Ando, Chiyo K. Imamura, Kenichi Yoshimura, Kentaro Yamazaki, Shuichi Hironaka, Kei Muro

**Affiliations:** 1 Division of Gastroenterology and Hepatology, Department of Internal Medicine, Keio University School of Medicine, 35 Shinanomachi, Shinjuku-ku, Tokyo, 160-8582 Japan; 2 Keio Cancer Center, Keio University School of Medicine, 35 Shinanomachi, Shinjuku-ku, Tokyo, 160-8582 Japan; 3 Center for Advanced Medicine and Clinical Research, Nagoya University Hospital, 65 Tsurumai-cho, Showa-ku, Nagoya, 466-8550 Japan; 4 Department of Clinical Pharmacokinetics and Pharmacodynamics, Keio University School of Medicine, 35 Shinanomachi, Shinjuku-ku, Tokyo, 160-8582 Japan; 5 Center for Integrated Medical Research, Hiroshima University Hospital, Hiroshima University, 1–2-3, Kasumi, Minami-ku, Hiroshima, 734-8551 Japan; 6 Department of Gastrointestinal Oncology, Shizuoka Cancer Center, 1007 Shimonagakubo, Nagaizumi-cho, Sunto-gun, Shizuoka, 411-8777 Japan; 7 Department of Medical Oncology and Hematology, Oita University Faculty of Medicine, 1–1 Idaigaoka, Hasama-machi, Yufu-shi, Oita 879-5593 Japan; 8 Department of Clinical Oncology, Aichi Cancer Center Hospital, 1–1, Kanokoden, Chikusa-ku, Nagoya-shi, Aichi Japan

**Keywords:** Advanced gastric cancer, Paclitaxel, Nab-paclitaxel, Peritoneal dissemination

## Abstract

**Background:**

Ramucirumab (RAM) with weekly paclitaxel (wPTX) is a standard second-line therapy for advanced or recurrent gastric cancer. Nanoparticle albumin-bound paclitaxel (nab-PTX), an albumin-bound form of PTX, was developed to improve the therapeutic index of taxane treatment. However, the ABSOLUTE trial showed the non-inferiority of weekly nab-PTX (w-nab-PTX) to wPTX with respect to overall survival (OS) as second-line therapy for advanced or recurrent gastric cancer, and subgroup analysis of patients with peritoneal dissemination showed favourable OS and progression-free survival (PFS) in the w-nab-PTX arm compared to those in the wPTX arm. This study evaluated whether w-nab-PTX plus RAM is more effective than wPTX plus RAM for patients with peritoneal dissemination.

**Methods:**

The P-SELECT trial (WJOG10617G) is a prospective, open-label, multicentre, randomised phase II study evaluating wPTX plus RAM (arm A) versus w-nab-PTX plus RAM (arm B). Key eligibility criteria include the following: 1) histologically proven adenocarcinoma, 2) unresectable or recurrent gastric cancer, 3) peritoneal dissemination, 4) intolerance or refractory to first-line therapy including fluoropyrimidines, and 5) ECOG Performance Status (PS) 0–2. Patients are randomised to either arm at a 1:1 ratio stratified by institution, PS, and severity of ascites. PTX (80 mg/m^2^; days 1, 8, and 15) and RAM (8 mg/kg; days 1 and 15) are administered every 4 weeks in arm A, while nab-PTX (100 mg/m^2^; days 1, 8, and 15) instead of PTX is administered in arm B. The primary endpoint is OS, and the main secondary endpoints are PFS, objective response rate, safety, neuropathy-specific quality of life, and biomarkers. To maintain a probability of ≥70% to ensure the hazard ratio for OS in arm B is lower than 0.90, 105 subjects are required. The study was initiated in October 2018 and is being conducted in 58 centres of the West Japan Oncology Group.

**Discussion:**

The results of this study will determine whether w-nab-PTX plus RAM has the potential to be a preferred therapeutic option for advanced and recurrent gastric cancer with peritoneal dissemination, compared to wPTX plus RAM.

**Trial registration:**

This study was prospectively registered in the Japan Registry of Clinical Trials (jRCTs031180022, October 1, 2018).

## Background

Gastric cancer (GC) is the fifth most common cancer globally and the third most deadly cancer [1]. In advanced GC (AGC), the peritoneum is one of the most common metastatic sites. Peritoneal dissemination is characterised by serious clinical symptoms such as massive ascites, bowel obstruction, hydronephrosis, and obstructive jaundice, which are associated with poor prognosis and quality of life (QOL). The current standard first-line therapy for unresectable advanced or recurrent GC is fluoropyrimidine plus either cisplatin [2] or oxaliplatin [3] for HER2-negative AGC, and the addition of trastuzumab to fluoropyrimidine plus platinum therapy [4] for HER2-positive AGC. In the second-line therapy, weekly paclitaxel (wPTX) plus ramucirumab (RAM) is regarded as standard therapy, irrespective of HER2 status, based on the results of two phase III trials, the WJOG4007 Trial [5] and the RAINBOW trial [6].

Nanoparticle albumin-bound paclitaxel (nab-PTX) is a formulation of PTX that binds to human serum albumin, forming nanoparticles. The pharmaceutical characteristics of nab-PTX are dependent on the binding of PTX, a highly water-insoluble compound, to human serum albumin, followed by the preparation of a freeze-dried formulation [7]. This enables its administration as a suspension in physiological saline without solvents (polyoxyethylene castor oil or ethanol) as required for traditional PTX [7], which makes it unnecessary to prophylactically administer steroids or antihistamines to prevent hypersensitivity. Other advantages of nab-PTX are reduced infusion time and its use for alcohol-sensitive patients.

In the ABSOLUTE trial, triweekly nab-PTX (tri-nab-PTX arm), weekly nab-PTX (w-nab-PTX arm), and weekly PTX (wPTX arm) were compared for patients with AGC who were refractory to first-line therapy, including fluoropyrimidines [8]. This study showed the non-inferiority of w-nab-PTX to wPTX in terms of overall survival (OS; hazard ratio [HR]: 0.97, 97.5% confidence interval [CI]: 0.76–1.23, non-inferiority one-sided *p* = 0.0085), which was a primary endpoint. A recent phase II study of RAM with w-nab-PTX therapy also showed sufficient safety and efficacy as a second-line treatment for GC [9]. The objective response rate (ORR) was 54.8% (90% CI: 41.0–68.0), the median progression-free survival (PFS) was 7.6 months (95% CI: 5.4–8.1), and the median OS was not reached at the time of data cut-off. Thus, w-nab-PTX plus RAM is regarded as a second-line treatment for GC patients [10].

Subgroup analysis of the ABSOLUTE trial showed significant benefits in selecting patients who are likely to respond to w-nab-PTX therapy. In patients with peritoneal dissemination, the HR for OS in the w-nab-PTX arm compared to the wPTX arm was 0.78 (95% CI: 0.59–1.03, interaction *p* = 0.011). Moreover, in patients with ascites, the HRs (95% CIs) for OS at each level of ascites (none, small, moderate, large) were 1.10 (0.48–1.48), 1.02 (0.71–1.48), 0.66 (0.38–1.17), and 0.25 (0.07–0.94), respectively. These findings suggest that w-nab-PTX might be more effective than wPTX for patients with peritoneal dissemination or moderate to large ascites. However, it is still unclear whether w-nab-PTX plus RAM is more effective than wPTX plus RAM, especially in certain subgroups of AGC patients. This is a randomised phase II trial of wPTX plus RAM versus w-nab-PTX plus RAM for unresectable AGC with peritoneal dissemination that is refractory to first-line therapy, including fluoropyrimidines.

## Methods/design

### Objectives

The objective of this study is to explore and evaluate the efficacy and safety of w-nab-PTX plus RAM as second-line therapy for progressing or recurring unresponsive/intolerant unresectable GC with peritoneal dissemination, using wPTX plus RAM as the control. The primary endpoint is OS. Secondary endpoints are PFS, ORR, disease control rate (DCR), time to treatment failure (TTF), proportion of ascites response, proportion of ascites control, safety, neuropathy-specific QOL, and biomarkers for nab-PTX and RAM.

### Study design

**Fig. 1:**
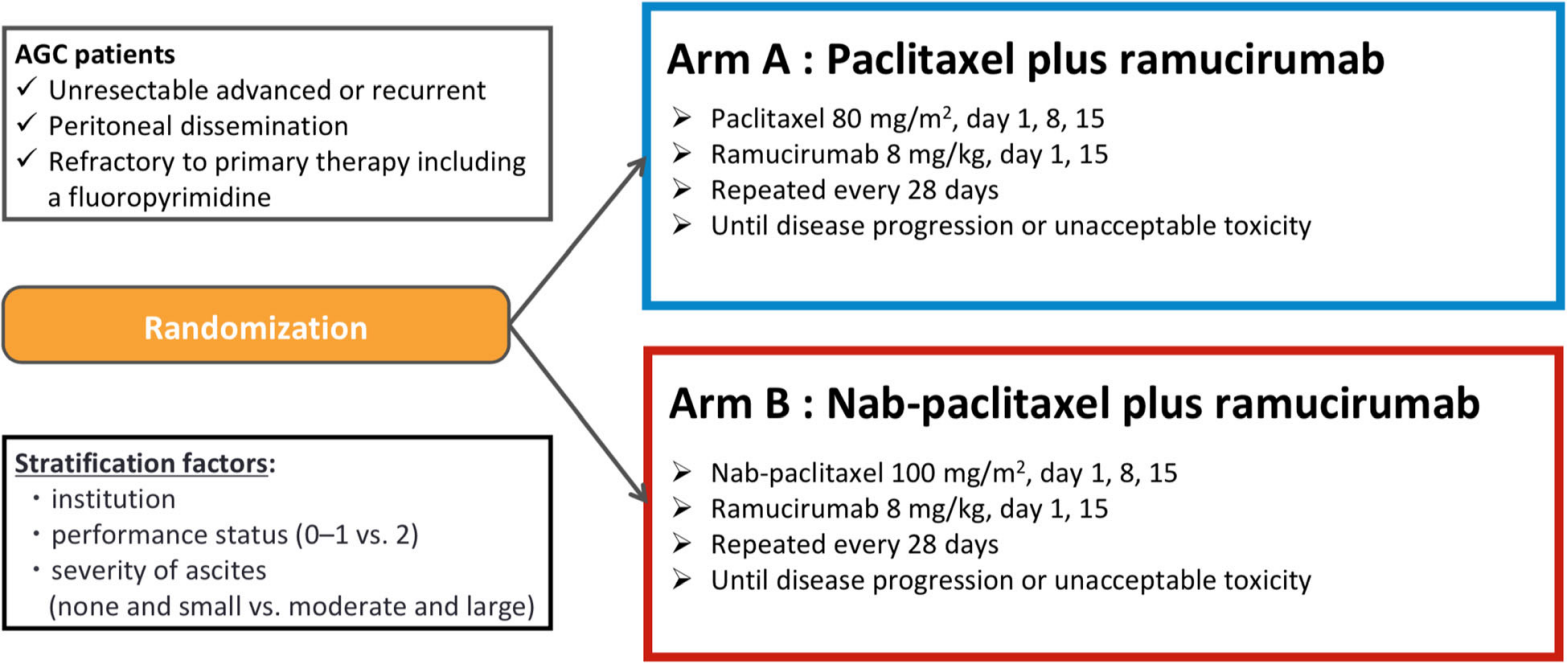
P-SELECT trial (WJOG10617G): flow chart

The P-SELECT trial (WJOG10617G) is a prospective, open-label, randomised, multicentre phase II study, which takes place in 58 centres of the West Japan Oncology Group (WJOG) in Japan (Fig. 1). The definitions for peritoneal dissemination are as follows: i) contrast enema/enterography: obvious malignant intestinal stenosis or deformation of the intestinal wall; ii) CT scan: obvious peritoneal mass or ascites, hydronephrosis that is not attributable to a cause other than peritoneal dissemination, increased density of peritoneal adipose tissue, thickening of the intestinal wall; iii) clinical signs: palpable metastasis in the pouch of Douglas, board-like rigidity of the abdomen that is not attributable to a cause other than peritoneal dissemination; iv) operative findings (including exploratory laparoscopy): lesions suggestive of peritoneal dissemination, confirmed by pathological diagnosis of peritoneal dissemination. Patients are randomised in a 1:1 ratio either to arm A or arm B. Randomisation is performed centrally with the minimisation method, with stratification for institution, ECOG Performance Status (PS, 0–1 vs. 2), and severity of ascites (none and small vs. moderate and large). Eligibility criteria are shown in Table 1. Key inclusion criteria include the following: 1) histological diagnosis of primary gastric or oesophagogastric junction adenocarcinoma, 2) unresectable or recurrent GC diagnosis, 3) patients with peritoneal dissemination, 4) intolerant or refractory to first-line therapy including fluoropyrimidines, 5) PS 0–2. Key exclusion criteria include the following: 1) multiple active cancers, 2) palliative ascites aspiration within the past 2 weeks, 3) grade 2 or worse peripheral sensory neuropathy, 4) patients with intestinal obstruction.

**Table 1:** Patient eligibility—inclusion and exclusion criteria

Inclusion criteria	Exclusion criteria
**1) Age ≥ 20 years** **2) Informed consent** **3) Histological diagnosis of primary gastric or oesophagogastric junction adenocarcinoma** **4) Unresectable gastric or oesophagogastric junction cancer, recurrent gastric, or oesophagogastric junction cancer** **5) At least one of i) to iv) listed below, which indicates peritoneal dissemination** **i) Contrast enema/enterography: obvious malignant intestinal stenosis or deformation of the intestinal wall** **ii) CT: obvious peritoneal mass or ascites, hydronephrosis not attributable to a cause other than peritoneal dissemination, increased density of peritoneal adipose tissue, thickening of the intestinal wall** **iii) Clinical signs: palpable metastasis in the pouch of Douglas, board-like rigidity of the abdomen not attributable to a cause other than peritoneal dissemination** **iv) Operative findings (including exploratory laparoscopy): lesions suggestive of peritoneal dissemination, confirmed by pathological diagnosis of peritoneal dissemination** **6) Patients without massive pleural effusion** **7) Patients without advanced central nervous system (CNS) metastases** **8) Patients with evaluable lesions (whether or not lesions are measurable is irrelevant)** **9) Intolerant or refractory to first-line therapy, including fluoropyrimidine** **10) Patient without history of using taxane or angiogenesis inhibitors** **11) Patients without history of radiotherapy including irradiation of the abdominal region** **12) ECOG performance status: 0, 1, or 2** **13) Adequate organ function**	1) Active multiple cancer (synchronous multiple cancer or metachronous multiple cancer where patient has been disease-free for less than 3 years); however, lesions corresponding to carcinoma in situ (intraepithelial carcinoma) or intramucosal cancer assessed as cured by local treatment are not classified as active multiple cancers. 2) History of sensitivity to any of the drugs used in this study (including alcohol or albumin sensitivity) 3) Patients with a history of surgery under general anaesthesia within 28 days 4) Patients who have had deep vein thrombosis, pulmonary embolism, or another major form of thromboembolism up to 12 weeks prior to enrolment 5) Palliative ascites aspiration (excluding diagnostic puncture for laboratory tests) within the past 2 weeks 6) Active infection requiring systemic treatment 7) Uncontrolled hypertension despite hypotensive drug therapy 8) Diabetes under treatment with continuous insulin use or uncontrolled diabetes 9) Patients with severe pulmonary disease 10) Unstable angina (attacks occurring or exacerbated within the past 3 weeks) or myocardial infarction within the past 3 months 11) Serious haemorrhagic disorder or vasculitis or an episode of serious gastrointestinal bleeding 12) Grade 2 or worse peripheral sensory neuropathy 13) Patients with liver cirrhosis (Child Pugh B or C) or previous hepatic encephalopathy 14) Patients with intestinal obstruction 15) Patients receiving continuous systemic administration of steroids 16) Patients receiving antiplatelet drugs 17) Mental illness or mental symptoms that would interfere with participation in the study 18) Pregnant, breast-feeding, a chance of pregnancy, or expecting to give birth 19) Patients deemed unsuitable as study subjects by an investigator for any other reason

### Procedures

Patients receive RAM (8 mg/kg) intravenously on days 1 and 15 plus PTX (80 mg/m^2^) intravenously on days 1, 8, and 15 in arm A or nab-PTX (100 mg/m^2^) intravenously on days 1, 8, and 15 in arm B. Three dose levels including the initial dose are set for dose reduction: 80, 70, and 60 mg/m^2^ for PTX; 100, 80, and 60 mg/m^2^ for nab-PTX; and 8, 6, and 5 mg/kg for RAM. In both groups, if grade 4 neutropenia or thrombocytopenia, grade 3 or worse febrile neutropenia, or grade 3 peripheral neuropathy is recorded, the dose of PTX or nab-PTX will be reduced to the next lower dose. If grade 3 infusion reactions or fatal PTX/nab-PTX-related adverse events are observed, PTX/nab-PTX will be discontinued. If patients have ≥2+ on a dipstick and ≥ 2 g/24 h proteinuria or if grade 3 RAM-related adverse events except hypertension and proteinuria are recorded, the dose of RAM will be reduced to the next lower dose. If patients have a grade 3 infusion reaction, ≥3 g/24 h proteinuria, or grade 4 hypertension, RAM will be discontinued. Study treatment will be repeated every 4 weeks until disease progression, unacceptable toxicities, or withdrawal consent. During study treatment, palliative radiotherapy and no other anti-cancer or investigational drugs are allowed.

### Outcomes

Tumour assessments using CT scans of the chest, abdomen, and pelvis are performed within 4 weeks before randomisation and are repeated every 8 weeks from randomisation to the discontinuation of protocol treatment. OS is defined as the time from randomisation to death from any cause. PFS is defined as the time from randomisation to disease progression or death from any cause. ORR will be assessed according to RECIST (version 1.1) only for patients with target lesions (complete or partial response). DCR will also be assessed according to RECIST irrespective of patients with target lesions (complete response, partial response, stable disease, or non-complete response/non-progressive disease). TTF is defined as the time from randomisation to progression, discontinuation of the study drug, or death from any cause. The proportion of ascites response and proportion of ascites control will be assessed only for patients with ascites (small, moderate, or large). The severity of adverse events will be graded according to the National Cancer Institute Common Terminology Criteria for Adverse Events (version 4.0).

### Sample size calculation and statistical analysis

By referring to the HR of the ABSOLUTE Trial in subjects with peritoneal dissemination [8], the expected HR for the present study was set to 0.80. To maintain a probability of 70% or higher so that the HR for OS in the w-nab-PTX plus RAM arm would be lower than 0.90, the required sample size for primary analysis was set to 50 patients per group. Therefore, study enrolment was set to 105 subjects to accommodate ineligible patients. The enrolment period has been set to 3 years. The follow-up period has been set to 1 year from enrolment of the last patient.

As a primary analysis, the HR of the w-nab-PTX plus RAM arm with respect to the wPTX plus RAM arm in terms of OS will be analysed in accordance with intention-to-treat principles. The HR and its 95% CI will be determined using the multivariate Cox proportional hazard model with adjustments for the stratification factors other than institutions. Median survival time will be determined, and its 95% CI will be calculated using the Brookmeyer-Crowley method. In addition, a stratified log-rank test using stratification factors other than institutions will be used for comparisons in both groups.

### Neuropathy-specific QOL assessment

With an evaluation of neurotoxicity as the primary objective, peripheral neuropathy onset conditions will be investigated for wPTX plus RAM and w-nab-PTX plus RAM groups using a Patient Neurotoxicity Questionnaire [11] and Functional Assessment of Cancer Therapy-taxane questionnaire [12] with health-related QOL for peripheral neuropathy; this is specially designed to evaluate taxane-based drugs. Furthermore, an examination will be conducted prior to treatment and then 2, 4, 6, 9, and 12 months after treatment inception. This will be followed by a summation of each QOL measurement item and comparison between two groups for all registered cases.

### Assessment of RAM pharmacokinetics

Peripheral vein blood will be obtained prior to RAM administration on the 15th day of cycle 1 to evaluate the plasma trough concentration (C_min_) of RAM.

### Correlative research

Secreted protein acidic and rich in cysteine (SPARC) and caveolin-1 (Cav-1) expression in tumour tissues will be evaluated as predictive biomarkers for efficacy. For tumour tissues, an endoscopic biopsy or surgical specimen obtained at the time of diagnosis (prior to the first chemotherapy treatment), can be used as the specimen. Separate consent will be obtained for correlative research.

### Study organisation

The WJOG is responsible for project management of the trial. The tasks of the WJOG include coordination of investigator meetings, monitoring, data management, and audits. Monitoring procedures will be adapted to the study-specific risks for patients. Regular on-site monitoring visits are not planned, but WJOG group rules will be regularly audited during this study.

### Data management, control of data consistency, and quality control

The investigator or a designated representative must enter all information required by the protocol in the electronic case report form after anonymisation to protect patient privacy. Automatic checks for data completeness, validity, and consistency will be programmed by the WJOG. The investigator or a designated representative is obliged to provide clarification or respond to queries when generated. Each dataset is checked for errors or inconsistencies before merging with data from the other data sources or time points via the assigned study number to create a comprehensive dataset. Data access is limited to the authors and the research assistants of the WJOG research team.

### Ethical aspects and trial registration

The P-SELECT trial (WJOG10617G) was approved by the Certified Review Board of Keio (CRB3180017) and has been prospectively registered in the Japan Registry of Clinical Trials (jRCT; jRCTs031180022, October 1, 2018, https://jrct.niph.go.jp/en-latest-detail/jRCTs031180022).

## Discussion

Despite the recent development of systemic chemotherapy, the prognosis remains poor for GC patients with peritoneal dissemination and even worse than that for other metastatic sites [13]. A significantly lower response rate to chemotherapy has been reported for GC patients with peritoneal dissemination [14], which is thought to be due to the presence of the “blood peritoneal barrier”, which isolates the peritoneal cavity from intravenous chemotherapy [15]. For these reasons, the prevention and treatment of peritoneal dissemination to improve survival remain a crucial clinical challenge.

The development of treatment strategies using formulations with a high potential to penetrate the peritoneum is required for patients with a poor prognosis owing to peritoneal dissemination. In a mouse peritoneal dissemination model, nab-PTX resulted in a significant decrease in ascites, exerted antiproliferative effects, and was associated with better overall prognosis when administered at equitoxic doses, compared to traditional PTX formulations [16]. Differences in drug formulations between nab-PTX and traditional PTX might explain the efficacy of w-nab-PTX [17]. Traditional PTX requires solvents such as Cremophor EL and ethanol, whereas nab-PTX is a solvent-free nanoparticulate formulation consisting of PTX and human serum albumin [7]. Nab-PTX is associated with increased uptake by utilising the endogenous albumin pathways of gp60- and Cav-1-mediated endovascular transcytosis and binding between albumin and SPARC, which enables the drug to more thoroughly permeate the tumour [18]. Therefore, molecules such as Cav-1 or SPARC could be tumour-penetrative biomarkers, as well as prognostic biomarkers, for nab-PTX. As a correlate to the indicated outcome measurements, molecular analysis of the albumin transport pathways related to nab-PTX transport into a tumour will be conducted in this study.

The results from the RAINBOW trial showed significantly longer OS in the wPTX plus RAM group than in the wPTX alone group; therefore, wPTX plus RAM is considered the standard for treating unresectable or recurrent GC, regardless of the presence or absence of peritoneal dissemination at the time of treatment [6]. In this study, 315 patients (47.4%) among 665 patients had peritoneal dissemination at the time of enrolment. The subgroup analysis of OS showed that wPTX plus RAM was better for patients with peritoneal dissemination (HR 0.807, 95% CI: 0.627–1.038) compared to that with wPTX alone. These results showed that RAM had additive effects to wPTX therapy, and it is expected that similar effects of RAM will be observed for patients with peritoneal dissemination treated with w-nab-PTX.

Gardner et al. reported that the proportion of activated (free) PTX in the blood increases with nab-PTX treatment compared to that with traditional PTX [19]. PTX inhibits angiogenesis by binding the vascular endothelium [20], but nab-PTX exhibits a higher rate of vascular endothelial binding than traditional PTX [21]. In addition, nab-PTX has shown significant vasopermeability and tissue penetrability relative to traditional PTX; these properties might further intensify the additional effects of RAM, which is a direct vascular endothelial growth factor receptor 2 (VEGFR2) antagonist. For these reasons, the present study will be able to elucidate the effect of adding RAM to traditional PTX or nab-PTX for subjects with peritoneal dissemination.

Plasma trough concentration of RAM will be evaluated in this study. A correlation between higher trough concentrations of RAM and longer OS has been reported in the RAINBOW and REGARD trials [22]. Therefore, it is necessary to assess RAM exposure to compare clinical outcomes between the two arms.

We will also perform a QOL evaluation of peripheral neuropathy. Peripheral neuropathy is well-known adverse event associated with chemotherapy for malignant tumours, and especially AGC. In AGC, platinum is frequently used as the first-line chemotherapy. Hence, caution must be exercised as peripheral neuropathy can be exacerbated when taxanes are used as secondary therapy. Because the worsening of peripheral neuropathy leads to the dose reduction, holding, or discontinuation of taxanes, its appearance and aggravation can significantly decrease a patient’s QOL and negatively impact patient prognosis. Recognising the differences in onset conditions for peripheral neuropathy in both traditional PTX and nab-PTX groups might be extremely beneficial as it improves a patient’s QOL and prognosis.

A previous single arm phase 2 trial suggested that w-nab-PTX plus RAM therapy is associated with promising activity and manageable toxicities [9]. The next emerging issue for the secondary treatment of gastric cancer is the question of whether wPTX plus RAM or w-nab-PTX plus RAM is better, as well as how to use this approach. If this trial proves that w-nab-PTX plus RAM is efficacious compared to wPTX plus RAM, the former could represent a new treatment option as a second-line chemotherapy for GC with peritoneal dissemination. Developing personalised therapeutic regimens classified by the presence or absence of peritoneal dissemination might also be a breakthrough for developing treatments for other malignant tumours.

## Supplementary information

**Additional file 1.** SPIRIT 2013 Checklist.

## Data Availability

Patient recruitment began in October 2018 and is ongoing. We plan to publish the results in a later report. Authorship will be handled according to standards set by the International Committee of Medical Journal Editors (http://www.icmje.org/recommendations/browse/roles-and-responsibilities/defining-the-role-of-authors-and-contributors.html). The SPIRIT checklist for the current study is available in Additional file 1.

## Abbreviations

Cav-1: caveolin-1
CI: confidence interval
CPT-11: irinotecan
DCR: disease control rate
ECOG: Eastern Cooperative Oncology Group
HR: hazard ratio
nab-PTX: nanoparticle albumin-bound paclitaxel
OS: overall survival
ORR: objective response rate
PFS: progression-free survival
PS: performance status
PTX: paclitaxel
QOL: quality of life
RAM: ramucirumab
SPARC: secreted protein acidic and rich in cysteine
TTF: time to treatment failure
VEGFR2: vascular endothelial growth factor receptor 2
